# ApoE variant p.V236E is associated with markedly reduced risk of Alzheimer’s disease

**DOI:** 10.1186/1750-1326-9-11

**Published:** 2014-03-10

**Authors:** Christopher W Medway, Samer Abdul-Hay, Tynickwa Mims, Li Ma, Gina Bisceglio, Fanggeng Zou, Shane Pankratz, Sigrid B Sando, Jan O Aasly, Maria Barcikowska, Joanna Siuda, Zbigniew K Wszolek, Owen A Ross, Minerva Carrasquillo, Dennis W Dickson, Neill Graff-Radford, Ronald C Petersen, Nilüfer Ertekin-Taner, Kevin Morgan, Guojun Bu, Steven G Younkin

**Affiliations:** 1 Department of Neuroscience, Mayo Clinic College of Medicine, Jacksonville, FL 32224, USA; 2 Division of Biomedical Statistics and Informatics, Mayo Clinic College of Medicine, Rochester, MN 55905, USA; 3 Department of Neurology, St. Olav’s Hospital, Edvard Griegs Gate 8, 7006 Trondheim, Norway; 4 Department of Neuroscience, Norwegian University of Science and Technology, NTNU, 7491 Trondheim, Norway; 5 Department of Neurodegenerative Disorders, Medical Research Centre, Polish Academy of Sciences, Warsaw, Poland; 6 Department of Neurology, Medical University of Silesia, Katowice, Poland; 7 Department of Neurology, Mayo Clinic College of Medicine, Jacksonville, FL 32224, USA; 8 Department of Neurology, Mayo Clinic College of Medicine, Rochester, MN 55905, USA; 9 Mayo Alzheimer Disease Research Center, Mayo Clinic College of Medicine, Rochester, MN 55905, USA; 10 Translational Cell Sciences – Human Genetics, School of Life Sciences, Queens’s Medical Centre, University of Nottingham, Nottingham NG7 2UH, UK

## Abstract

Recent genome-wide association studies (GWAS) of late-onset Alzheimer’s disease (LOAD) have identified single nucleotide polymorphisms (SNPs) which show significant association at the well-known *APOE* locus and at nineteen additional loci. Among the functional, disease-associated variants at these loci, missense variants are particularly important because they can be readily investigated in model systems to search for novel therapeutic targets. It is now possible to perform a low-cost search for these “actionable” variants by genotyping the missense variants at known LOAD loci already cataloged on the Exome Variant Server (EVS). In this proof-of-principle study designed to explore the efficacy of this approach, we analyzed three rare EVS variants in *APOE*, p.L28P, p.R145C and p.V236E, in our case control series of 9114 subjects. p.R145C proved to be too rare to analyze effectively. The minor allele of p.L28P, which was in complete linkage disequilibrium (*D’* = 1) with the far more common *APOE* ϵ4 allele, showed no association with LOAD (*P* = 0.75) independent of the *APOE* ϵ4 allele. p.V236E was significantly associated with a marked reduction in risk of LOAD (*P =* 7.5×10^−05^; OR = 0.10, 0.03 to 0.45). The minor allele of p.V236E, which was in complete linkage disequilibrium (*D’* = 1) with the common APOE ϵ3 allele, identifies a novel LOAD-associated haplotype (*APOE* ϵ3b) which is associated with decreased risk of LOAD independent of the more abundant *APOE* ϵ2, ϵ3 and ϵ4 haplotypes. Follow-up studies will be important to confirm the significance of this association and to better define its odds ratio. The ApoE p.V236E substitution is the first disease-associated change located in the lipid-binding, C-terminal domain of the protein. Thus our study (i) identifies a novel *APOE* missense variant which may profitably be studied to better understand how ApoE function may be modified to reduce risk of LOAD and (ii) indicates that analysis of protein-altering variants cataloged on the EVS can be a cost-effective way to identify actionable functional variants at recently discovered LOAD loci.

## Introduction

The international effort to catalog common variants [minor allele frequency (MAF) > 5%] in the human genome (HapMap Project [1]) paved the way for genome-wide association studies (GWAS), which have proven to be a powerful tool for understanding the genetics of complex diseases. GWAS of late-onset Alzheimer’s disease (LOAD), a genetically complex disease with an estimated 60-80% heritability [2], have identified common SNPs which reach genome-wide significance at the well-known *APOE* locus and at nineteen additional loci. The identification of these common GWAS SNPs that replicably associate with LOAD is a significant breakthrough, but it is important to recognize that these SNPs do not identify the functional disease-modifying variant(s) to which they are linked, and they do not fully account for LOAD heritability. It is now clear that at least some of this missing heritability is accounted for by rare variants with large effect size. This is well-illustrated by the recently discovered rare, LOAD-associated missense variants in the *TREM2* gene [3,4]. Importantly, this locus was not detected using the GWAS approach because the *TREM2* LOAD-associated variants, which are not included in GWAS genotyping arrays, are too rare to be detected at genome-wide significance by analysis of the common GWAS SNPs to which they are linked.

Among the functional variants at GWAS loci, those that alter proteins are particularly important because they can readily be investigated in model systems to search for novel therapeutic targets. The Exome Variant Server (EVS, http://evs.gs.washington.edu/EVS/) catalogs whole exome sequencing of 4300 unrelated European Americans, a series large enough to detect virtually all exonic variants with a minor allele frequency (MAF) of 0.1% (1/1000) or more. Thus expensive resequencing is no longer required to discover such variants, and it is now possible to perform a meaningful, low-cost search for “actionable” variants with MAF > 0.1% by genotyping protein-altering variants cataloged on the EVS in large European American case-control series. To evaluate the utility of this approach, we searched the EVS for protein-altering *APOE* variants with MAF > 0.1% and found just two, p.L28P (0.17%) and p.V236E (0.12%) in European Americans. Both were analyzed in our large European American case control series of 4128 LOAD subjects and 4986 non-demented controls (Table 1). In addition we analyzed one extremely rare variant, p.R145C (0.026%), that did have a MAF > 1% in African Americans.

**Table 1:** Sample demographics for case-control series

**Series**	**n (%)**				**Mean AAD ± SD (Years)**				**Females (%)**				**ϵ4+ Subjects (%)**			
	**AD**		**CON**		**AD**		**CON**		**AD**		**CON**		**AD**		**CON**	
Jacksonville	1020	(41.2)	1453	(58.8)	77.7	(6.46)	79.5	(7.86)	623	(61.1)	838	(57.7)	653	(64.0)	338	(23.3)
Rochester	600	(19.9)	2409	(80.1)	80	(7.72)	78.3	(5.56)	363	(60.5)	1294	(53.7)	328	(54.7)	571	(23.7)
Poland	250	(100)	0	(0)	74.4	(5.19)	NA	(NA)	156	(62.4)	NA	(NA)	139	(55.6)	NA	(NA)
Norway	345	(38.5)	552	(61.5)	80.2	(7.25)	75.4	(6.73)	241	(69.9)	330	(59.8)	217	(62.9)	132	(23.9)
NCRAD	702	(77.1)	209	(22.9)	75.2	(6.76)	78.3	(8.88)	455	(64.8)	129	(61.7)	551	(78.5)	34	(16.3)
Autopsy*	1211	(76.9)	363	(23.1)	81.4	(8.56)	75.9	(8.14)	695	(57.4)	155	(42.7)	744	(61.4)	98	(27.0)
Total	4128	(45.3)	4986	(54.7)	78.7	(7.76)	78.2	(6.91)	2533	(61.4)	2746	(55.1)	2632	(63.8)	1155	(23.2)

*Autopsy controls unlike the clinical controls, who were neurologically normal, include some non-AD degenerative disorders.

## Result

In this proof of principle study, we used our large LOAD case-control series (Table 1) to analyze three missense variants in the *APOE* gene that were mined from the EVS database: rs769452 (T/c, p.L28P), rs769455 (C/t, p.R145C) and rs199768005 (T/c, p.V236E). Comparison of EVS European Americans with the control subjects in our series showed no significant difference in the MAFs for rs769452 (*P* = 0.27), rs769455 (*P =* 0.46) or rs199768005 (*P* = 0.075).

rs769455 (ApoE p.R145C) was successfully genotyped in 3955 AD cases and 4590 controls. With only 4 heterozygotes in the AD cases, 1 in the control group, and no homozygotes, p.R145C was too rare to analyze effectively as expected from its EVS frequency. Analysis by a Fisher’s exact test yielded an odds ratio (OR) and 95% confidence interval (95% CI) of 4.64 (0.52 to 41.56) with a p value of 0.13. In African Americans, the MAF for rs769455 on the EVS is 1.39% as compared to 0.026% in European Americans, so we evaluated this variant in our African American LOAD case control series of 168 LOAD patients and 333 non-demented control subjects. There were 9 heterozygotes in the AD cases compared to 17 in the control group and no homozygotes. A chi-square test showed no evidence of allelic association with LOAD (*P* = 0.91: OR = 1.05, 0.46 to 2.38), but the small series tested has relatively little statistical power as an OR of approximately 3.3 is required for 80% power to detect association at α = 0.05. Analysis in additional case-control studies is clearly needed to evaluate the association of this rare variant with LOAD.

rs769452 (ApoE p.L28P) was successfully genotyped in 2996 late-onset AD cases and 3951 control samples. There were 36 heterozygotes in the AD cases compared to 20 in the control group and no homozygotes. Analysis of rs769452 by a Fisher’s exact test showed significant (*P =* 1.6×10^−03^) association with increased risk of LOAD (OR = 2.39, 1.38 to 4.37). In African Americans (AA), the MAF for rs769452 on the EVS is 0.023% as compared to 0.17% in European Americans, so this variant was not genotyped in our small AA series.

rs199768005 (ApoE p.V236E) was successfully genotyped in 4128 late-onset AD cases and 4986 control samples. There were 2 heterozygotes in the AD cases compared to 23 in the control group and no homozygotes. Confirmatory genotyping using a custom TaqMan assay was 100% concordant. Analysis of rs199768005 by a Fisher’s exact test showed significant (*P =* 7.5×10^−05^) association with markedly reduced risk of LOAD (OR = 0.10, 0.03 to 0.45). rs199768005 was not genotyped in our small AA series, as its minor allele was never detected in the much larger set of 2203 EVS AA subjects.

The well-known *APOE* ϵ2, ϵ3, and ϵ4 haplotypes are formed by two *APOE* missense SNPs, rs429358 (T/c, p.C112R) and rs7412 (C/t, p.R158C), as shown in Table 2. The minor alleles of rs429358 and rs7412 tag the ϵ4 and ϵ2 haplotypes respectively; the ϵ3 haplotype has major alleles at both loci. Haplotype phasing showed that the minor allele of rs199768005 (p.V236E) is in phase (*D’ =* 1) with *APOE* ϵ3 (major alleles at rs429358 and rs7412) and that the minor allele of rs769452 (ApoE p.L28P) is in phase with *APOE* ϵ4 (minor allele at rs429358, major at rs7412). Thus p.V236E occurs on the ϵ3 backbone subdividing the ϵ3 haplotype into *APOE* ϵ3b (minor allele of rs199768005) and *APOE* ϵ3a (major allele of rs199768005) whereas p.L28P subdivides ϵ4 into *APOE* ϵ4b (minor allele of rs769452) and *APOE* ϵ4a (major allele of rs769452), as shown in Table 2. Univariate logistic regression using an additive model with sex and age at diagnosis as covariates gave results for the ϵ3b (OR = 0.11, 0.02 to 0.36; *P* = 2.32×10^−03^) and e4b (OR = 2.49, 1.45 to 4.41; *P* = 1.17×10^−3^) haplotypes which were essentially identical to the Fisher exact results for the missense variants that tag them. As expected, univariate logistic regression showed that the ϵ4 allele was associated with significant, markedly increased risk of AD and that the ϵ2 and ϵ3a alleles were associated with significant, markedly reduced risk. To determine whether *APOE* ϵ3b or ϵ4b are significantly associated with LOAD independent of the ϵ2, ϵ3, and ϵ4 alleles, we performed multivariate logistic regression using a model that included not only sex and age at diagnosis as covariates but also the *APOE* ϵ4 and ϵ2 alleles, with ϵ3a as referent (Table 2). When the *APOE* ϵ4 and ϵ2 alleles were included as covariates, the ϵ4b showed no association (*P* = 0.75), indicating that the minor allele of p.L28P does not significantly modify the risk associated with *APOE* ϵ4 when it is present on that haplotype (Table 2). Importantly, the ϵ3b allele contributed significantly (OR = 0.10, 0.02 to 0.35; *P* = 2.16×10^−3^) to a model that included *APOE* ϵ2 and ϵ4 as covariates with *APOE* ϵ3a as referent. Thus, compared to *APOE* ϵ3a, *APOE* ϵ3b (ApoE p.236E) is associated with a significantly decreased risk of AD that is independent of the ϵ2 and ϵ4 alleles.

**Table 2:** ***APOE *** **Haplotypes formed by three variants and their association with AD**

***APOE Haplotype†***	**rs429358**		**rs7412**		**rs199768005**		**rs769452**		**Minor allele frequency (%)**		Logistic regression*			
	**p.C112R**		**p.R158C**		**p.V236E**		**p.L28P**				**Univariate**		**Multivariate**	
	**Base**	**AA**	**Base**	**AA**	**Base**	**AA**	**Base**	**AA**	**AD**	**CON**	**OR**	**P**	**OR**	**P**
ϵ2 allele	T	Cys	t	Cys	T	Val	T	Leu	3.15%	8.50%	0.35 (0.30-0.40)	<2x10^−16^	0.46 (0.38-0.54)	<2x10^−16^
ϵ3a allele	T	Cys	C	Arg	T	Val	T	Leu	58.0%	79.0%	0.35 (0.32-0.37)	<2x10^−16^	1.00 (REF)	REF
ϵ3b allele	T	Cys	C	Arg	c	Glu	T	Leu	0.024%	0.23%	0.11 (0.02-0.36)	2.32x10^−03^	0.10 (0.02-0.35)	2.16x10^−03^
ϵ4a allele	C	Arg	C	Arg	T	Val	T	Leu	37.4%	12.3%	5.00 (4.52-5.50)	<2x10^−16^	4.80 (4.35-5.30)	<2x10^−16^
ϵ4b allele	C	Arg	C	Arg	T	Val	c	Pro	0.62%	0.26%	2.49 (1.45-4.41)	1.17x10^−03^	0.91 (0.51-1.66)	0.75

Alleles in uppercase denote a major allele, alleles in lower case denote a minor allele.

*Logistic regression models corrected for sex and age-at-diagnosis, and assume an additive effect.

† Haplotype phasing showed that the minor allele of rs199768005 (p.V236E) is in phase (*D’ =* 1) with the major alleles at rs429358 and rs7412, indicating that it occurs on the ϵ3 backbone thereby subdividing the ϵ3 haplotype into *APOE* ϵ3b (minor allele of rs199768005) and *APOE* ϵ3a (major allele of rs199768005). rs769452 (p.L28P) subdivides ϵ4 into *APOE* ϵ4b (minor allele of rs769452) and *APOE* ϵ4a (major allele of rs769452).

## Discussion

Our results show that ApoE p.V236E occurs on the *APOE* ϵ3 backbone creating a rare *APOE* ϵ3b haplotype, which is significantly associated with LOAD independent of the *APOE* ϵ2, ϵ3, and ϵ4 alleles. Comparison of the 95% CI for *APOE* ϵ3b (OR = 0.10*, 0.02 to 0.35*) with that for *APOE* ϵ2 (OR = 0.46, *0.38 to 0.54*), indicates that, in our series, the ϵ3b allele reduced risk of AD as much or more than the *APOE* ϵ2 allele (Table 2, Multivariate Logistic Regression). In this regard, it is worth noting that, of the 2 LOAD patients carrying p.V236E, one developed dementia at an advanced age (98 yrs, *APOE* ϵ3a/ϵ3b genotype) and the other, who was diagnosed at 68, also carried an ϵ4 allele (*APOE* ϵ3b/ϵ4 genotype), which likely counters the protection afforded by p.V236E. The 23 non-demented control carriers included 7 with ages of 64-88 years with ϵ3b/ϵ4 genotypes, 14 with ages of 68-91 with ϵ3b/ϵ3a genotypes, and 2 with ages of 68 and 92 with ϵ3b /ϵ2 genotypes. To verify the significance of the association observed in our series and to improve the OR estimate for p.V236E, replication in a similarly large series will be important, ideally a series with GWAS genotypes that can be used to adjust for the potentially confounding effect of population stratification. If *APOE* ϵ2 and ϵ3b act similarly, as seems likely, then analysis of the functional effects of ϵ2 as compared to the novel ϵ3b allele identified here could provide insight into the common or distinct mechanism whereby they reduce risk of LOAD.

In three previous studies [5-7], rs769452 (ApoE p.L28P) was genotyped in a total of 2630 subjects (1329 AD/1401 Control: 1118/1123 [5], 117/121 [6], 93/157 [7]. These studies also found that ApoE p.L28P occurs on the *APOE* ϵ4 backbone. The risk associated with the minor allele of rs769452, which tags the rare *APOE* ϵ4b allele, appeared to be greater than the risk of *APOE* ϵ4 in two of these studies [5,7] but less in the other study [6]. When the results from these previous series were combined with those presented here, the OR for *APOE* ϵ4 vs. all other alleles was 4.31 (3.96 to 4.70) as compared to 4.04 (2.74 to 6.00) when *APOE* ϵ4b was compared to the same referent group. Thus the combined results from all series, like those from our series alone (Table 2), indicate that the minor allele of p.L28P does not substantially modify the risk associated with *APOE* ϵ4 when it is present on that haplotype. Replication in additional large series will be important to confirm this finding.

ApoE is a 299 amino acid long protein with a highly hydrophobic lipid binding domain in the C-terminal region, and a receptor binding domain in the N-terminal region. Bridged by a protease sensitive hinge region, the N- and C-terminal domains appear to interact when ApoE is delipidated, preventing lipoprotein receptor docking and internalization of unlipidated ApoE [8]. The two missense variants that create the *APOE* ϵ2 (p.C112R) and *APOE* ϵ4 (p.R158C) alleles both alter amino acids in the N-terminal region, which may interfere with receptor binding. The missense variant (p.V236E) that creates the *APOE* ϵ3b allele is the first LOAD-associated variant to alter a C-terminal amino acid [9]. The protein encoded by *APOE* ϵ3b has previously been described as APOE*2 [10] because upon isoelectric focusing it migrates similarly to the APOE2 protein encoded by the *APOE* ϵ2 allele. Studies of individuals carrying p.V236E have found no lipoprotein abnormalities [11]. Pathogenicity prediction using SIFT and PolyPhen-2 both suggest p.V236E is damaging, substituting a nonpolar, hydrophobic valine for the negatively charged, hydrophilic glutamic acid. Position 236 is proximal to the lipid binding domain (244-272) and interestingly it is located within a region believed to be important for ApoE oligomerization (230-243) [12]. The substitution of a hydrophobic valine for an ionic glutamic acid is consistent with p.V236E altering the lipid binding property of ApoE, or affecting aggregation. Additionally, in light of the interaction between ApoE N- and C-terminal domains, p.V236E could alter ApoE folding and receptor binding. We are currently investigating these possibilities.

In this proof of principle study, we searched the EVS for protein-altering *APOE* variants with MAF > 0.1% and found just two, p.L28P (0.17%) and p.V236E (0.12%). Both were tested for association with LOAD in our large case-control series, and one (p.V236E) was significantly associated with markedly decreased risk of LOAD, independent of the *APOE* ϵ2, ϵ3, and ϵ4 alleles. It will now be important to determine if this same cost-effective approach can be used to identify additional LOAD-associated, protein altering variants in genes at any of the recently discovered LOAD loci that might profitably be investigated to identify novel therapeutic targets for AD.

## Materials and methods

### Case-control subjects

Demographic information on the LOAD patients and non-demented control subjects that were analyzed is shown in Table 1. Approval was obtained from the ethics committee or institutional review board of each institution responsible for the ascertainment and collection of samples. Written informed consent was obtained for all individuals who participated in this study.

The Mayo case-control series consists of European Americans ascertained at the Mayo Clinic Jacksonville, Mayo Clinic Rochester, and in the Mayo Clinic autopsy-confirmed samples (Autopsy in Table 1). Additional Caucasian subjects from the United States were obtained through the National Cell Repository for Alzheimer’s Disease (NCRAD in Table 1), and European Caucasian subjects were obtained from Norway [13] and Poland [14,15]. All subjects in the Mayo clinical case-control series were diagnosed by a neurologist at the Mayo Clinic in Jacksonville, Florida, or Rochester, Minnesota. The neurologist confirmed a Clinical Dementia Rating score of 0 for all Jacksonville and Rochester subjects enrolled as controls; cases had diagnoses of possible or probable AD made according to NINCDS-ADRDA criteria [16]. Clinical LOAD cases and controls in the NCRAD, Polish, and Norwegian were ascertained similarly. In the autopsy-confirmed series, all brains were evaluated by Dr. Dennis Dickson and came from the brain bank he maintains at the Mayo Clinic in Jacksonville, FL. In the Autopsy series the diagnosis of definite AD was also made according to NINCDS-ADRDA criteria. Only samples with an age-at-diagnosis (AAD) above 60 years, with sex and *APOE* covariates (ϵ2, ϵ3, ϵ4 alleles) available, were included in this study.

### Nomenclature

To conform to most of the literature on ApoE, our numbering of ApoE residues begins with the first amino acid that remains after removal of the 18 amino acid leader sequence. This is different from EVS numbering which begins with the first amino acid in the leader sequence [17]. The protein encoded by the *APOE* ϵ3b allele, which is created by the minor allele of p.V236E (see Table 2), has previously been described as APOE*2 [10,11] because upon isoelectric focusing it migrates similarly to the APOE2 protein encoded by *APOE* ϵ2 allele.

### Genotyping

*APOE* missense variants resulting in p.L28P (rs769452), p.R145C (rs769455) and p.V236E (rs199768005) were genotyped using SEQUENOM’s MassArray iPLEX technology (SEQUENOM Inc, San Diego, CA, USA). SEQUENOM’s Typer Analyzer 4.0 was used to conduct off machine processing and genotype calling. Confirmatory genotyping of p.V236E was carried out using a custom TaqMan assay in an ABI PRISM 7900HT Sequence Detection System with 384-Well Block Module (Applied Biosytems, California, USA). TaqMan assays were also employed to genotype the *APOE* missense variants resulting in p.R158C (rs7412) and p.C112R (rs429358) in order to identify the well-known APOE ϵ2, ϵ3, and ϵ4 alleles. Cluster calling was carried out using SDS software v2.2.3 (Applied Biosytems, California, USA). All Sequenom and TaqMan probe sequences are available on request.

### Statistical analysis

Analysis of control subjects using PLINK [18] (http://pngu.mgh.harvard.edu/~purcell/plink/), showed that all variants were in Hardy Weinberg equilibrium (P > 0.80). Allelic association was evaluated using Fisher’s exact method in PLINK. Haplotypic analysis was performed using the haplo.stats package in the R programming language (v2.14.1). Logistic regression was carried out adjusting for sex and age at diagnosis.

## Competing interests

The authors declare that they have no competing interests.

## Authors’ contributions

Study design was developed by SGY, GB, CWM, KM, NET and OAR. Variant discovery and preparatory bioinformatics analysis was performed by CWM and SGY. SAH, TM, LM, FZ and CWM carried out genotyping. CWM, SGY and SP carried out the statistical analysis. SGY, NET, MC, GB, LM, JS, SBS, JOA, MB, ZKW, DWD, NGR and RCP were involved in sample acquisition and/or DNA preparation. The manuscript was drafted by CWM, SGY and SAH. All authors read and approved the final manuscript.
